# Folate and Cobalamin Serum Levels in Healthy Children and Adolescents and Their Association with Age, Sex, BMI and Socioeconomic Status

**DOI:** 10.3390/nu13020546

**Published:** 2021-02-07

**Authors:** Paulina Kreusler, Mandy Vogel, Anja Willenberg, Ronny Baber, Yvonne Dietz, Antje Körner, Uta Ceglarek, Wieland Kiess

**Affiliations:** 1 LIFE Leipzig Research Center for Civilization Diseases, University of Leipzig, 04103 Leipzig, Germany; Mandy.Vogel@medizin.uni-leipzig.de (M.V.); Ronny.Baber@medizin.uni-leipzig.de (R.B.); Ydietz@life.uni-leipzig.de (Y.D.); Antje.koerner@medizin.uni-leipzig.de (A.K.); uta.ceglarek@medizin.uni-leipzig.de (U.C.); wieland.kiess@medizin.uni-leipzig.de (W.K.); 2 Hospital for Children and Adolescents and Center for Pediatric Research (CPL), University of Leipzig, 04103 Leipzig, Germany; 3 Institute of Laboratory Medicine, Clinical Chemistry and Molecular Diagnostics (ILM), University Hospital Leipzig, 04103 Leipzig, Germany; Anja.willenberg@medizin.uni-leipzig.de

**Keywords:** children, adolescents, folate, cobalamin, body mass index, socioeconomic status, reference values, population-based

## Abstract

This study proposes age- and sex-specific percentiles for serum cobalamin and folate, and analyzes the effects of sex, age, body mass index (BMI), and socioeconomic status (SES) on cobalamin and folate concentrations in healthy children and adolescents. In total, 4478 serum samples provided by healthy participants (2 months–18.0 years) in the LIFE (Leipzig Research Centre for Civilization Diseases) Child population-based cohort study between 2011 and 2015 were analyzed by electrochemiluminescence immunoassay (ECLIA). Continuous age-and sex-related percentiles (2.5th, 10th, 50th, 90th, 97.5th) were estimated, applying Cole’s LMS method. In both sexes, folate concentrations decreased continuously with age, whereas cobalamin concentration peaked between three and seven years of age and declined thereafter. Female sex was associated with higher concentrations of both vitamins in 13- to 18-year-olds and with higher folate levels in one- to five-year-olds. BMI was inversely correlated with concentrations of both vitamins, whilst SES positively affected folate but not cobalamin concentrations. To conclude, in the assessment of cobalamin and folate status, the age- and sex-dependent dynamic of the respective serum concentrations must be considered. While BMI is a determinant of both vitamin concentrations, SES is only associated with folate concentrations.

## 1. Introduction

The essential vitamins cobalamin and folate play an interdependent role in key cellular processes, namely nucleic acids synthesis, cell division, red blood cell formation, and nervous system myelination [1,2,3]. It is therefore important that special attention be paid to the maintenance of an adequate folate and cobalamin status during childhood and adolescence, which constitute critical periods of particular vulnerability, due to the higher nutrient requirements of rapid growth and physical, psychological, and neurocognitive development. For decades, extensive research has focused on the increased risks of adverse health effects related to suboptimal cobalamin and folate concentrations. Indeed, folate deficiency is an established risk factor for neural tube defects [1,3], while cobalamin deficiency is associated with dementia in the elderly and impaired cognitive and neurological function such as neuropathy and demyelination [2,3]. Both vitamins have been linked to failure to thrive, psychomotor developmental delay, megaloblastic anemia, and hyperhomocysteinemia, which has been recognized as an independent risk factor for cardiovascular disease [1,2,3]. Recent literature recognizes that risk factors for lifestyle-related noncommunicable diseases often originate in childhood and progress into adulthood [4]. In this context, dietary changes offer an opportunity to effectively reduce the predisposition for health risks associated with cobalamin and folate deficiency and to establish health-conscious behavior early in life. In order to correctly assess the folate and cobalamin biomarker status in children and implement necessary dietary interventions, valid reference values are required. However, large studies addressing optimal serum concentrations in healthy European pediatric populations are still scarce and, to date, universally accepted cut-offs have not been established for either adults or children. Commonly applied reference values for serum cobalamin and folate concentrations were established in adults or in the USA [3,5], where food fortification with folic acid is mandatory and dietary habits differ substantially from those in Europe. The transferability of these reference values to pediatric populations in Europe is therefore limited. While several studies have been carried out on folate and cobalamin status in European children [6,7,8,9,10,11,12,13,14,15,16,17,18,19], only four focused on reference values for healthy children and/or adolescents (Table S1) [6,7,8,9]. At present, to the best of the authors’ knowledge, no single study exists which is not limited by age range, small sample size, or the publication of only mean/median values for broad age groups.

The present study seeks to establish population-based age- and sex-dependent distributions for serum cobalamin and folate, deriving these values from a large pediatric cohort study on this topic, which consisted of 4478 cases involving healthy young Germans aged between 2 months and 18 years. Further, it seeks to investigate the impact of age, sex, body mass index (BMI; BMI = body weight/(height^2^) in kg/m^2^), and socioeconomic status (SES) on the respective vitamin serum levels.

## 2. Materials and Methods

### 2.1. Study Design

The data processed and analyzed in this study was collected within the context of the LIFE (Leipzig Research Centre for Civilization Diseases) Child Study. Since 2011, LIFE Child—a rolling prospective, longitudinal, population-based cohort study with a life-course approach to health and disease—has sought to investigate the influence of genetic, metabolic, and environmental factors on growth, development, and health from pregnancy to adulthood. Additional details regarding the study design are provided elsewhere [20,21]. The LIFE Child cohort has served to establish pediatric reference ranges in prior studies, e.g., regarding liver enzymes [22] and cystatin C [23].

### 2.2. Ethical Review and Study Registration

The LIFE Child study (NCT02550236) was designed in conformity with the Declaration of Helsinki and its later amendments [24]. The study protocol and procedures were approved by the Ethical Committee of the University of Leipzig (Reg. No. 264-10-19042010). Written informed consent was obtained from all parents or legal guardians and children over the age of 12 [21].

### 2.3. Participants and Setting

Subjects were recruited through collaboration with kindergartens, schools, local hospitals, outpatient clinics, and health centers. The eligibility criteria required participants to be aged between 2 months and 18 years and without chronic, chromosomal, and syndromal diseases. At each visit, a multi-professional team gathered data via clinical examinations, questionnaires, interviews, and samples of biological materials such as blood, urine, and hair. For subjects enlisted during the first year of life, data was collected at 3, 6, and 12 months of age [20,21]. For the part of the study discussed here, all samples with valid measurements of serum folate or cobalamin from 2011 to 2015 were included. The final dataset consisted of 4478 samples from 2467 children and adolescents aged between 2 months and 18 years (Figure S1).

### 2.4. Variables and Data Sources/Measurements

Prior to venous blood collection using serum and EDTA monovettes (Sarstedt AG&Co, Nümbrecht, Germany), children aged ≥ 4 years were asked to fast overnight for at least 8 h. However, failure to comply was not regarded as an exclusion criterion as serum cobalamin is relatively independent of recent intake [2], and fasting is presumably non-essential when assessing the folate status of populations [1]. After the samples had undergone preanalytical processing in the LIFE laboratory, they were promptly sent to be analyzed at the Institute of Laboratory Medicine, Clinical Chemistry and Molecular Diagnostics within the University Hospital Leipzig. Electrochemiluminescence immunoassays (ECLIA) were carried out according to the manufacturer’s protocol for the in vitro quantitative determination of serum folate and cobalamin (Cobas 8000 e602, Roche Diagnostics GmbH, Mannheim, Germany), with measuring ranges of 1.36–45.4 nmol/L and 36.9–1476 pmol/L respectively. Weight was measured to an accuracy of 0.05 kg with the “Seca 701” scale (seca GmbH & Co. KG, Hamburg, Germany). A stadiometer (“Prof. Keller”; Längenmesstechnik GmbH Limbach, Limbach-Oberfrohna, Germany) with 0.1 cm graduation was used to measure height. The children’s weight status was assessed by transforming the calculated BMI values into standard deviation scores (SDS) based on age- and sex-based German reference data [25]. Subsequently, the following weight groups were defined as underweight (BMI-SDS < −1.28), normal-weight (−1.28 ≤ BMI-SDS < 1.28), overweight (1.28 ≤ BMI-SDS < 1.88), and obese (BMI-SDS ≥ 1.88) according to the German guidelines [26]. SES was assessed using the Winkler Index, which is calculated on the basis of the equivalized disposable household income, and the educational attainment and occupational status of both parents. The sum of these scores produces a total score between 3 and 21 (3–8.4 = low, 8.5–15 = middle, 15.5–21 = high SES) [27].

### 2.5. Statistical Analysis

Data management, statistical analysis, and visualization were performed utilizing the statistical software R [28]. A *p*-value of < 0.05 was considered statistically significant. All values were subjected to plausibility testing. No outliers were eliminated from the data set. Age- and sex-dependent percentile curves for folate and cobalamin were estimated applying Cole’s LMS-type method. The percentile curves were then smoothed and modelled as a function of age under the assumption of a Box-Cox power exponential distribution of the target variables to compensate for random percentile fluctuations [29]. A resampling method was used to account for multiple measurements per child/family [21]. In children younger than 1.5 years, 43.2% (*n* = 192) of folate measurements were higher than the upper limit of quantification. Therefore, a right-censored Box-Cox-t distribution was assumed for the calculation of folate values. Measurement values of both vitamins were then transformed into age- and sex-adjusted standard deviation scores (SDS). Univariate and multivariate hierarchical linear regression analyses were employed to determine relations between parameters (age, sex, BMI-SDS, SES) and the SDS for folate and cobalamin. All models were tested and adjusted for interactions between covariates. Due to the high percentage of right-censored values, censored regression was applied when modelling associations between folate SDS and covariates. Here, the number of measurements per child was used as weights to control for repetitive measurements per child. To assess differences in vitamin levels between sexes, non-transformed values were used.

## 3. Results

### 3.1. Description of the Study Population

Of the initial sample, 4478 cases representing 2448 participants were included. Table 1 summarizes the characteristics of the final study cohort stratified by age-groups. The cohort consisted of 76.6% normal-weight, 7.3% underweight, 7.2% overweight, and 7.9% obese participants. Overall, the sample approximates to the BMI distribution in German children and adolescents [30]. The middle SES group made up more than half (57.2%) of the cohort. Participants of low SES (10.3%) were underrepresented, whereas children of high SES (27.3%) were overrepresented [27].

### 3.2. Age- and Sex-Specific Reference Intervals of Serum Folate and Cobalamin Based on Generally Healthy Children

**Figure 1:**
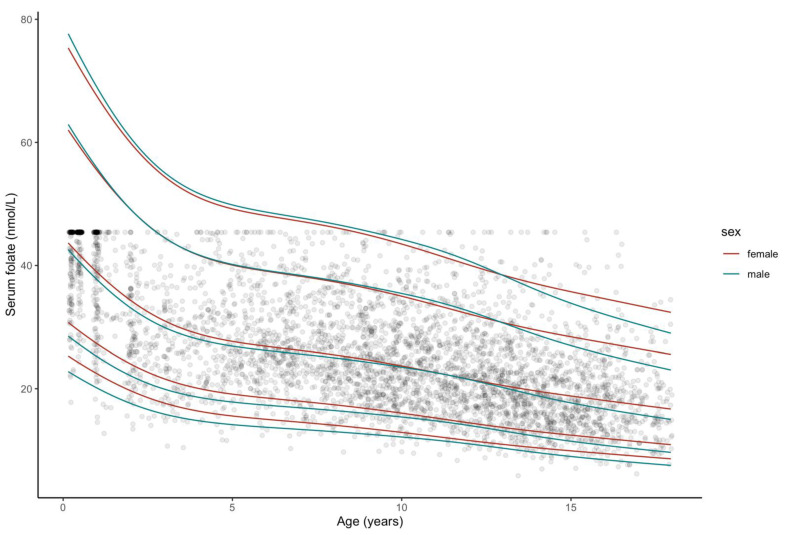
Smoothed percentile curves for serum folate concentrations according to age, based on censored data from a sample of generally healthy participants from the LIFE (Leipzig Research Centre for Civilization Diseases) Child study population. In total, there are 4171 observations from participants aged 2 months–18 years, single data points depicted. From bottom: 2.5th (P2.5),10th (P10), 50th (P50, median), 90th (P90), 97,5th (P97.5) percentiles are marked. Smoothed percentiles produced with Cole’s LMS-type method.

**Figure 2:**
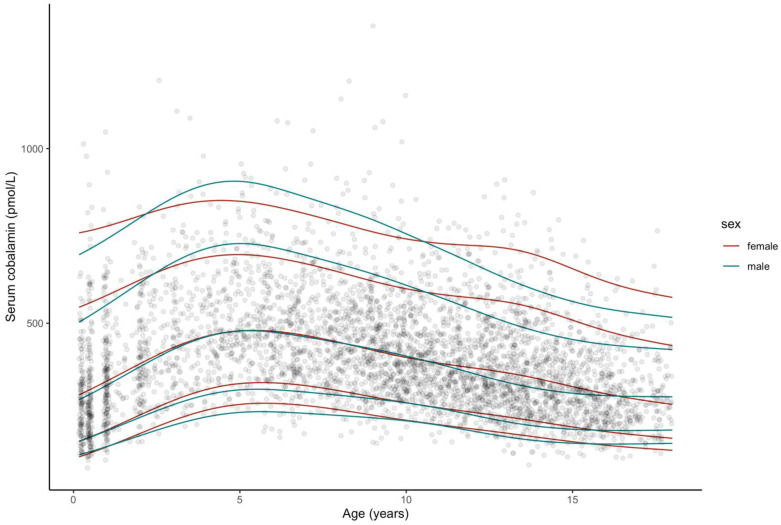
Smoothed percentile curves for serum cobalamin concentrations according to age based on a sample of generally healthy participants from the LIFE Child study population. In total, there are 4229 observations from participants aged 2 months–18 years, single data points depicted. From bottom: 2.5th (P2.5), 10th (P10), 50th (P50, median), 90th (P90), 97.5th (P97.5) percentiles are marked. Smoothed percentiles produced with Cole’s LMS-type method.

The smoothed percentile curves for serum folate and cobalamin are illustrated in Figure 1 and Figure 2. Corresponding tables of reference intervals stratified by age and sex for both vitamins are provided in Supplementary Tables S2 and S3.

Figure 1 illustrates the age-specific patterns in folate concentration levels. There is a clear continuous downward trend with increasing age in both sexes. The slope is steepest during the first four years of life. Thereafter, a gentler decline is observed. At age 13, the median percentiles of folate concentration in males and females intersect, indicating a slightly steeper fall in concentrations in teenage boys. The range between the 2.5th and the 97.5th percentiles is largest during infancy and decreases thereafter. Both intra- and inter-age group comparisons found large variances in measurements.

Figure 2 reveals a sharp increase in cobalamin concentrations during the first five years of age, with the 97.5th percentile peaking at 906.54 pmol/L. Thereafter, cobalamin levels decline rapidly. While girls tend to present higher cobalamin levels following birth and during the first year of life, in the upper percentiles, concentrations subsequently rise more rapidly in boys. Passing the 15-year age mark, the slope of decline flattens markedly. Similar to folate, measurements vary widely between ages and within age-groups.

### 3.3. Sex, BMI-SDS and SES as Potential Determinants of Serum Folate and Cobalamin Status

**Figure 3:**
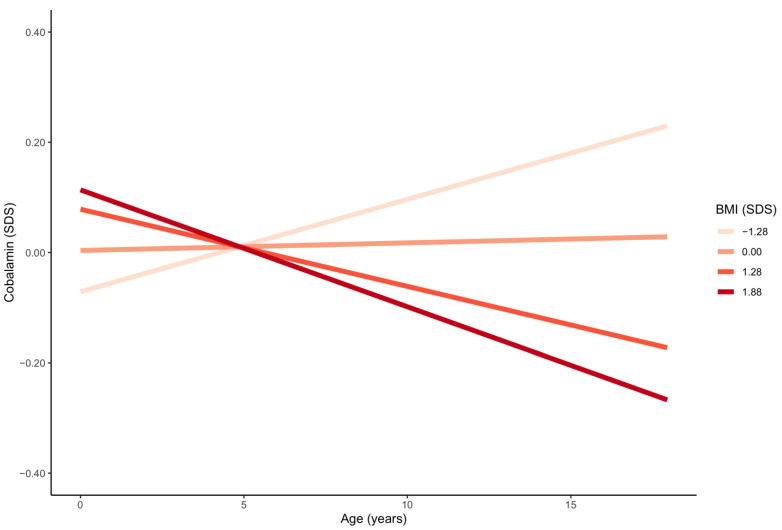
Age trends in serum cobalamin for four different values of BMI-SDS. BMI-SDS groups: underweight: BMI-SDS < −1.28; normal weight: BMI-SDS −1.28 to <1.28; overweight: BMI-SDS > 1.28 to 1.88; obese: BMI-SDS > 1.88. SDS = standard deviation score; BMI-SDS = body mass index standard deviation score.

**Figure 4:**
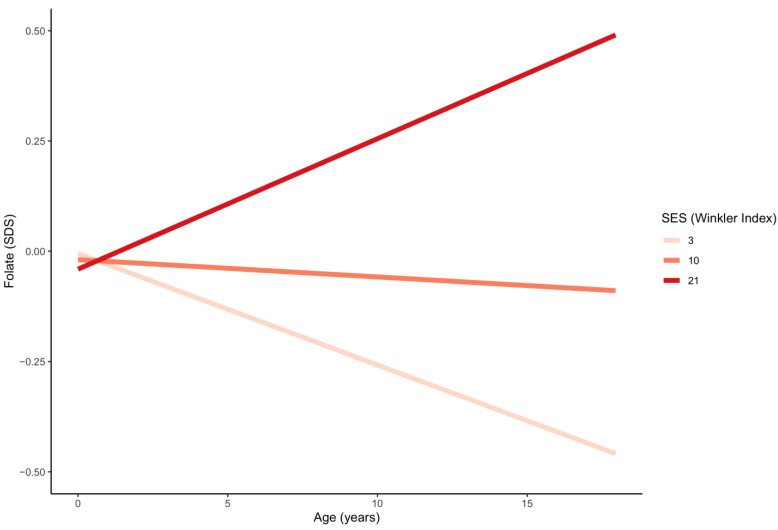
Age trends in serum folate for three different values of SES. SES groups: low: WI 3−8.4; middle: WI 8.5−15; high: 15.5−21. SES =socioeconomic status; WI = Winkler Index; SDS = standard deviation score.

Table 2 and Table 3 detail the results of the intercorrelation analysis. An inverse correlation was identified between BMI-SDS and folate (*p* < 0.001) and cobalamin (Figure S2). The association between cobalamin (but not folate) and BMI-SDS grew stronger with increasing age (*p* < 0.001) (Figure 3). Higher SES was significantly associated with higher folate levels (Figure S3). The effect of SES on folate increased with age (*p* < 0.001) (Figure 4). No significant relationship between SES and cobalamin was detected (*p* = 0.308). Age-dependent sex differences are best deduced from the provided percentiles. Multivariate regression indicated that females compared to males presented with higher mean folate levels between the ages of 1–5 years (*p* = 0.001) and 13–18 years (*p* < 0.001), and with higher mean cobalamin levels between the ages of 13–18 years (*p* < 0.001).

## 4. Discussion

Given the dependence of serum folate and cobalamin levels on dietary habits, mainly European studies were considered for literature comparison. Nonetheless, studies still lack consistency in terms of research purpose, the health, ethnic and socioeconomic status of subjects, geographical location, observed age ranges, inclusion and exclusion criteria, sample size, and methodology.

### 4.1. Comparison of Our Novel Reference Values with Previously Reported Concentrations

The folate and cobalamin concentrations in the present report were in reasonable accordance with earlier European pediatric studies [6,7,8,9,10,11,12,16,18,19]. As stated in the Results section, there was great variability of measurements not only between ages but also within each age group. In our study, folate values exceeded previously reported concentrations by 18–60%, especially in teenagers [7,10,11,13,15,16,17]. In contrast, only two previous studies found substantially higher folate values for some age groups [14,16]. In the case of one study, those higher folate levels may be at least partly explained by the voluntary folic acid fortification in Ireland [14]. The implications of food fortification on folate levels become apparent when evaluating data from the US, where folate concentrations almost tripled after the introduction of mandatory folic acid fortification in 1998 [31]. The mean levels in US children are approximately 1.5 to 3 times higher than those found in the current study [32,33]. European laboratories frequently apply reference ranges derived from a pre-fortification US pediatric cohort, ranges that appear to be set severely low when compared to data from this cohort [5]. The WHO suggested—on the basis of a sample of US adults—age-independent cut-offs of <10 nmol/L as indicative of folate deficiency [3]. In the current study, folate levels < 10 nmol/L were observed in merely 1.3% of cases (*n* = 53), a low prevalence compared to other studies. For instance, in Norway, 5.8% of two-year-old [11] and 13.2% of four- to six-year-old children [15] presented with levels below the cut-off, while in this study no child aged ≤ 6 years had folate levels < 10 nmol/L. Similarly, 6% of 6-to 15-year-old Greeks had levels below 7.7 nmol/L [12], compared to 0.1% (*n* = 2) of children of the same age in the current study. In the HELENA study, 15% of 12.5- to 17.45-year-olds had levels < 10.2 nmol/L [7], as opposed to 4.1% (*n* = 50) of same-aged teenagers in this cohort. Although the healthy adolescents in this study presented with relatively high folate levels compared to those of other European studies, the 2.5th percentile of 14- to 18-year-old males and 15- to 18-year-old females were <10 nmol/L.

As mentioned, overall, our cobalamin values compared well to previous findings, although values were 25–40% higher for some age groups [10,13] and, notably, 20–50% lower during infancy [6,12,13,15,16,17]. On the basis of data from an US adult population, the WHO defines cobalamin deficiency as a plasma cobalamin concentration (<150 pmol/L) for all ages [3]. In our study, values < 150 pmol/L were reported in only 1.3% of cases (*n* = 55). Approximately half of these (*n* = 28) were aged 12 months or younger. Thus, if we apply the WHO-recommended cut-off, 10% of infants would be categorized as cobalamin-deficient. By comparison, in the aforementioned Norwegian study, none of the children between 4–6 years of age had levels < 148 pmol/L [15], which ties well with this sample, where no children in the same age range presented with levels < 148 pmol/L. Similar to folate, the 2.5th percentile for cobalamin in both children aged ≤ 1 year and 16.5- to 18-year-old females was below the proposed cut-off.

Given the healthy status of subjects in this cohort, further research is warranted to explore if either the cut-offs recommended by the WHO are too high for the aforementioned age groups or if folate and cobalamin deficiency are alarmingly prevalent among otherwise healthy children and adolescents, particularly among females of childbearing age.

### 4.2. Comparison of Trends in Concentrations with Previous Findings

A key finding of this study was the steady decline of folate levels with increasing age, which supports the findings of several published studies in healthy European pediatric populations [7,8,9,10,12,16,17,19]. The peak levels in infants are likely attributable to the typically folate-rich breast milk and breast milk substitutes. Breast milk folate concentrations culminate at two to three months of lactation and decrease slightly thereafter [34]. Decreasing serum concentrations during the first years of life may be explained by the weaning from folate-rich breast milk and changes in gastrointestinal pH and bacterial flora caused by the introduction of solid foods [9,35]. Folate levels decline further during adolescence. This period is generally characterized by an incongruity between the amplified nutrient requirements due to accelerated growth, and the prevalence of unhealthy eating patterns such as irregular meals, snacking and increased consumption of unhealthy products [36,37]. Previous research has suggested that only half of European adolescents consume the recommended amount of fruits and vegetables and fewer than two thirds consume the recommended amount of milk products, while the intake of fats, meat, and sweets exceed recommendations [36].

As regards cobalamin, earlier studies are in accordance with either the aforementioned initial rise [9], the subsequent drop in concentrations [6,7,8,10,12,17,19], or both [13,16]. A possible explanation for the low concentration in young children may be the relatively poor cobalamin levels in breastmilk, which, contrary to folate, decrease during the first three to four months of lactation and are largely affected by the maternal cobalamin status [34]. In a Norwegian study, serum cobalamin levels decreased during the first 6 months, then gradually increased, surpassing birth concentrations at 24 months in breastfed children. In contrast, in non-breastfed infants, serum cobalamin increased steadily from birth until 24 months postpartum [9]. Other studies have also confirmed that breastfed infants tend to have lower cobalamin levels compared to infants fed with formula, which is typically high in cobalamin [2,34]. Cobalamin uptake during infancy may be further limited by an immature intrinsic factor system [38]. Nonetheless, it is assumed that during the first year of life the hepatic cobalamin stores are adequate to ensure appropriate growth and development [39], although intervention studies are needed to elucidate whether the low cobalamin levels in infants reflect a clinically relevant deficiency or should be considered a physiological variance. The rise and peak of cobalamin levels between the ages of three to seven years reported here and by others [13,16] follow the establishment of a solid diet and a decelerated growth rate in toddlers. Comparably to folate, the decreasing cobalamin concentrations during adolescence are likely attributable to the aforementioned incongruity between nutrient requirements and intake. After the age of 16 years, cobalamin levels plateaued in males, while in females concentrations declined continuously, albeit with a gentler slope than before. This confirms the results of the HELENA study, which reported gradually increasing values after the age of 15 years in both sexes [7]. Similarly, the KiGGS study reported a steady decrease in cobalamin levels in girls between 3 and 17 years of age, with levels stabilizing in boys from the age of 15 years onward [6].

Summarizing, our results support the notion that the age-related trends in folate and cobalamin concentrations are, to a great extent, associated with changes in eating patterns and nutritional requirements throughout childhood and adolescence.

### 4.3. Influence of Sex

As discussed above, we identified notable age-dependent sex differences with both vitamins. Female sex was significantly associated with higher concentrations of both vitamins in 13- to 18-year-olds, and with higher folate levels in 1- to 5-year-olds. The majority of previous studies conducted in European pediatric populations [10,11,12,13,17,19] identified no sex differences regarding serum concentrations of either vitamin, although a British study found higher folate levels in boys than in girls [8]. With respect to cobalamin concentrations, the HELENA study also reported higher levels in female teenagers compared to male ones [7]. The inconsistent findings may be partly explained by the limited detectability of sex differences in smaller studies, particularly when covering a relatively large age span. Altogether, the sex differences may suggest that, on the whole and compared to teenage boys, teenage girls in this study may have followed a more favorable diet, associated with a higher vitamin intake.

### 4.4. Influence of BMI

In this study, subjects with higher BMI tended to exhibit lower folate and cobalamin levels, which concurs with Huemer et al. [10]. In general, previous research has identified obese children as being at a higher risk of cobalamin deficiency [40]. These findings are supported by multiple studies reporting malnutrition associated with overweight and obesity in children, due to poor dietary content, and repeated short-term restrictive diets combined with increased nutritional requirements, leading to vitamin and mineral deficiencies [41,42,43]. Since cobalamin and folate play a pivotal role in metabolism, special attention should be paid not only to calorie reduction but also to promoting a nutrient-dense diet in overweight and obese children to ensure sufficient growth and development.

### 4.5. Influence of SES

Another finding was the association between higher SES and higher folate concentrations. To date, the impact of SES on serum folate has received surprisingly little attention, particularly in regard to children. While the present findings differ from those of a Greek study [12], they corroborate those of the HELENA study [44] and imply an association between SES-related nutritional habits and vitamin intake. This is consistent with research reporting that the consumption of foods containing folate—vegetables for the most part—is higher among upper SES subjects [44,45]. Curiously, the association between SES and folate levels was weakest in the youngest children, which might be explained by the elaborate mechanisms ensuring optimal folate supply in infancy, as discussed above. The current data highlight the importance of establishing a comprehensive evidence base to facilitate policies and lifestyle interventions as part of public initiatives to reduce health disparities.

Interestingly, cobalamin levels were not associated with SES in the current study. Although, these results are in line with certain previous studies [12], the HELENA study found lower serum cobalamin in females with lower maternal SES but not in males with lower maternal SES [44]. Similarly, a review of European studies found conflicting results regarding associations between SES and cobalamin intake in children and adolescents [45]. A possible explanation for the lack of association in this study may be that cobalamin is almost exclusively obtained by consuming animal products. There is a growing body of research suggesting that, in Germany, high meat-consumption is linked to lower SES, while people with high SES are more likely than people with low SES to follow a vegetarian diet [46,47]. Regarding cobalamin intake, this may diminish the aforementioned association that generally seems to exist between SES and diet quality/vitamin intake.

### 4.6. Strengths, Limitations, and Suggestions for Further Research

The study was limited by the underrepresentation of subjects from low SES families and non-white subjects, compared to the general population. Further, we did not consider the hemolysis index of blood samples, which in a few cases might have led to higher in vitro folate measurements. Moreover, the comparability of measures across different studies using different methods still poses some difficulties [1,2]. Most of the mentioned studies investigated cobalamin and folate levels via either protein-binding assays [6,7,12,16,18,19], as used in this study, via chromatography-based tandem mass spectrometry assays [8,17], or via microbiological assays [9,11,13,14,15]. While each diagnostic approach has its advantages and drawbacks, the protein-binding assay utilized in this study is widely available, rapid, high-throughput, automated, and relatively cost-effective, thereby meeting the requirements for clinical settings [1]. However, an apparent disadvantage is the limited serum folate measuring range. In the current study 6.1% (*n* = 254) of cases were above the maximum detection limit at 45.4 nmol/L. To overcome this drawback, a censored data approach was applied. Moreover, it should be noted that in the case of suspected cobalamin deficiency, serum cobalamin measurement should be combined with the assessment of functional biomarkers (methylmalonic acid (MMA) and/or total homocysteine) to confirm metabolic deficiency. Nonetheless, serum cobalamin continues to be the first-line test in a stepwise diagnostic approach, especially if deficiency screening is to be reasonable from a health economic perspective [48].

The LIFE Child study population constitutes a large population-based pediatric cohort of healthy young Europeans, thus facilitating the establishment of reference values. The large sample size is suitable for mapping the age-dependent dynamics of biomarkers, which is impossible to achieve in studies with a more modest number of participants. Moreover, the estimated percentiles reflect physiological dynamics, and thus avoid an arbitrary age-interval classification. Additionally, highly standardized data capture and laboratory procedures and continuous quality control ensure high reliability.

While the proposed age- and sex-specific reference values could be employed in a clinical context starting immediately, continued research is desirable in order to validate the suggested biomarker cut-offs in healthy European pediatric populations. Additionally, determination of reference values for other associated biomarkers (e.g., holo-transcobalamin, MMA, total homocysteine, and erythrocyte folate) to establish a greater degree of accuracy when interpreting the adequacy of folate and cobalamin status is needed. Moreover, the precise underlying mechanisms of the age- and sex-dependent dynamics in folate and cobalamin concentrations remain to be elucidated. This study has identified male sex, low SES, adolescence, and high BMI as factors associated with an increased risk of low folate and/or cobalamin serum concentrations. There is growing evidence regarding the complex and seemingly paradoxical relationship between pediatric obesity and food insecurity [49,50,51], defined as “limited or uncertain availability of nutritionally adequate and safe foods or limited or uncertain ability to acquire acceptable foods in socially acceptable ways” [52]. Household food insecurity contributes to unhealthy diet-related behaviors, increasing the risk of nutritional inadequacy and adiposity, particularly among older children [49,50,51]. Evidence regarding younger children remains inconclusive [50]. Further research is warranted on this subject to increase effectiveness of intervention strategies aimed at promoting healthy nutrition.

## 5. Conclusions

To conclude, this study contributes to a better understanding of cobalamin and folate concentrations by providing respective age- and sex-specific reference values derived from a healthy population-based pediatric cohort. When measuring cobalamin and folate levels in young people, these must be interpreted with regard to age and sex. While SES is associated with folate but not cobalamin concentrations, BMI and sex are determinants of the serum concentrations of both vitamins.

## Figures and Tables

**Table 1:** Description of the LIFE (Leipzig Research Centre for Civilization Diseases) Child cohort stratified by age intervals.

Age (Years)	0–1 (*n* = 453)	1–5 (*n* = 868)	6–12 (*n* = 1982)	13–18 (*n* = 1175)	Total (*n* = 4478)
Sex Category					
Male	243 (53.6%)	450 (51.8%)	1046 (52.8%)	560 (47.7%)	2299 (51.3%)
Female	210 (46.4%)	418 (48.2%)	936 (47.2%)	615 (52.3%)	2179 (48.7%)
Cobalamin (pmol/L)					
Mean (SD)	322 (161)	467 (165)	430 (145)	331 (122)	401 (155)
Median (Min, Max)	279 (86.4, 1050)	446 (131, 1200)	408 (135, 1350)	308 (95.1, 874)	377 (86.4, 1350)
Missing	92 (20.3%)	66 (7.6%)	52 (2.6%)	39 (3.3%)	249 (5.6%)
Folate (nmol/L)					
Mean (SD)	40.3 (6.76)	29.8 (8.60)	24.8 (7.62)	19.3 (6.88)	25.6 (9.45)
Median (Min, Max)	44.2 (17.8, 45.4)	28.7 (10.4, 45.4)	23.8 (8.34, 45.4)	18.2 (5.87, 45.4)	23.9 (5.87, 45.4)
Missing	103 (22.7%)	81 (9.3%)	70 (3.5%)	53 (4.5%)	307 (6.9%)
BMI-SDS					
Underweight	38 (8.4%)	44 (5.1%)	164 (8.3%)	83 (7.1%)	329 (7.3%)
Normal weight	380 (83.9%)	749 (86.3%)	1468 (74.1%)	833 (70.9%)	3430 (76.6%)
Overweight	32 (7.1%)	44 (5.1%)	142 (7.2%)	106 (9.0%)	324 (7.2%)
Obese	2 (0.4%)	14 (1.6%)	197 (9.9%)	143 (12.2%)	356 (7.9%)
Missing	1 (0.2%)	17 (2.0%)	11 (0.6%)	10 (0.9%)	39 (0.9%)
SES (WI)					
Low SES	39 (8.6%)	71 (8.2%)	199 (10.0%)	152 (12.9%)	461 (10.3%)
Middle SES	258 (57.0%)	500 (57.6%)	1136 (57.3%)	668 (56.9%)	2562 (57.2%)
High SES	130 (28.7%)	270 (31.1%)	564 (28.5%)	259 (22.0%)	1223 (27.3%)
Missing	26 (5.7%)	27 (3.1%)	83 (4.2%)	96 (8.2%)	232 (5.2%)

BMI-SDS groups: underweight: BMI-SDS < −1.28; normal weight: BMI-SDS −1.28 to <1.28; overweight: BMI-SDS > 1.28 to 1.88; obese: BMI-SDS > 1.88. SES groups: low: WI 3–8.4; middle: WI 8.5–15; high: 15.5–21. SD = standard deviation; Min = lowest value; Max = highest value; *n* = number of observations; BMI-SDS = body mass index standard deviation score; SES = socioeconomic status; WI = Winkler Index.

**Table 2:** Multivariate regression analysis results: age, body mass index (BMI), and socioeconomic status (SES).

Variable	Folate				Cobalamin			
	Estimate	Std. Error	*t*-Value	*p*-Value	Estimate	Std. Error	*t*-Value	*p*-Value
Age	−0.036	0.009	−3.846	<0.001	0.001	0.002	0.587	0.557
SES	0.012	0.004	2.830	0.005	0.003	0.003	1.019	0.308
BMI-SDS	−0.089	0.010	−8.701	<0.001	−0.001	0.015	−0.084	0.933
SES: age	0.003	0.001	4.679	<0.001				
BMI-SDS: age					−0.012	0.002	−5.632	<0.001

All calculations adjusted for sex, age, BMI-SDS, and SES. Age was set at 5 years for analysis. Folate and cobalamin SDS scores used for calculations. SDS = standard deviation score; BMI-SDS = body mass index standard deviation score; SES = socioeconomic status.

**Table 3:** Multivariate regression analysis results: sex stratified by age.

Age Interval (y)	Folate				Cobalamin			
	Estimate (β)	Std. Error	*t*-Value	*p*-Value	Estimate	Std. Error	*t*-Value	*p*-Value
0–1	0.538	0.486	1.107	0.269	19.930	11.569	1.723	0.085
1–5	1.406	0.431	3.265	0.001	3.561	7.821	0.455	0.649
6–12	0.270	0.230	1.174	0.240	5.748	4.338	1.325	0.185
13–18	1.383	0.289	4.791	<0.001	40.602	4.665	8.703	<0.001
0–18	0.703	0.172	4.094	<0.001	14.479	3.213	4.506	<0.001

After partition into age intervals, the analysis was carried out with non- transformed folate and cobalamin values. All calculations adjusted for age, BMI-SDS, and SES. Non- transformed folate and cobalamin values used for calculations. BMI-SDS = body mass index standard deviation score; SES = socioeconomic status.

## Data Availability

The data presented in this study are available on request from the corresponding author. The data are not publicly available, due to their containing information that could compromise the privacy/consent of research participants.

## Supplementary Materials

The following are available online at https://www.mdpi.com/2072-6643/13/2/546/s1, Table S1: A range of the published pediatric reference intervals for serum folate (SF) and cobalamin (B12), Table S2: Percentile distribution of plasma folate (nmol/L) as a function of age (0–18 years) derived from censored data based on the LIFE Child cohort of generally healthy children and adolescents (*n* = 4171), Table S3: Percentile distribution of plasma cobalamin (pmol/L) as a function of age (0–18 years) based on the LIFE Child cohort of generally healthy children and adolescents (*n* = 4229), Figure S1: Composition of the generally healthy pediatric reference population from the LIFE Child cohort, Figure S2: (a) Cobalamin, (b) folate according to BMI, Figure S3: Folate according to SES.
