# Supplementary material for: Folate and Cobalamin Serum Levels in Healthy Children and Adolescents and Their Association with Age, Sex, BMI and Socioeconomic Status
Source: Nutrients. 2021 Feb 7;13(2):546. doi: 10.3390/nu13020546 (PMC7915137; doi:10.3390/nu13020546)
Supplement: Supplementary file 1 [file nutrients-13-00546-s001.pdf]

Supplementary Materials

Table S1. A range of the published pediatric reference intervals for serum folate (SF) and cobalamin (B12)

Study	KiGGS [6]	HELENA [7]	NDNS [8]	Hay et al [9]
Year	2009	2011	2008	2008
Country	Germany	9 European countries	United Kingdom	Norway
Population	Population sample	Population sample	Population sample	Children delivered at Aker University Hospital (Oslo, Norway)
Age (years)	3-17.5	12.5-17.45	4-18	birth-24 mo
Sample size (cases)	B12: 13.050	1051	PF: 840, B12=770	1091
Study type, Exclusion criteria	Cross-sectional study, no exclusions	Cross-sectional study, inability to speak language, participation in another trial	Cross-sectional study, no exclusions	Longitudinal study, non-singleton births, gestational period <37w or >43w, birth weight > 2.5th percentile
Assay	ECLIA (Elecsys E2010, Roche, Mannheim, Germany)	CLIA (Immulite 2000, DPC Biermann GmbH, Bad Nauheim, Germany)	MEIA (Abbott IMx)	MA (colistin sulfate–resistant strain of Lactobacillus leichmannii (B12) and L. casei (SF))
Method	sex- and age-specific percentiles estimated using an LMS-type method	sex- and age-specific percentiles estimated using an LMS-type method	sex- and age-specific percentiles	Age-specific reference intervals for folate and cobalamin status according to breastfeeding status
Results	3rd, 5th, 10th, 25th, 50th, 75th, 90th, 95th, and 97th percentile (continuously with age: 3-17.5 y)	2.4th, 5th,10th, 25th, 50th, 75th, 90th, 95th, and 97.5th percentile (continuously with age: 12.5-17.45 y)	5th, 50th, 90th percentile (4-10y, 11-14y,15-18 y), stratified by sex for age 15-18 y	5th, 50th, 95th percentile (birth, 6 mo, 12 mo, 24 mo)

KiGGS nationwide health survey in children and adolescents carried out by the Robert Koch Institute, Germany; HELENA Healthy Lifestyle in Europe by Nutrition in Adolescence; NDNS National Diet and Nutrition Survey (NDNS); w = weeks; mo = month; y = years; ECLIA = electrochemiluminescence immunoassay; CLIA = chemiluminescent competitive immunoassay; MEIA = microparticle enzyme immunoassay; MA = microbiological assays; LMS = L skewness, M median, S coefficient of variation.

Figure S1. Composition of the generally healthy pediatric reference population from the LIFE Child cohort

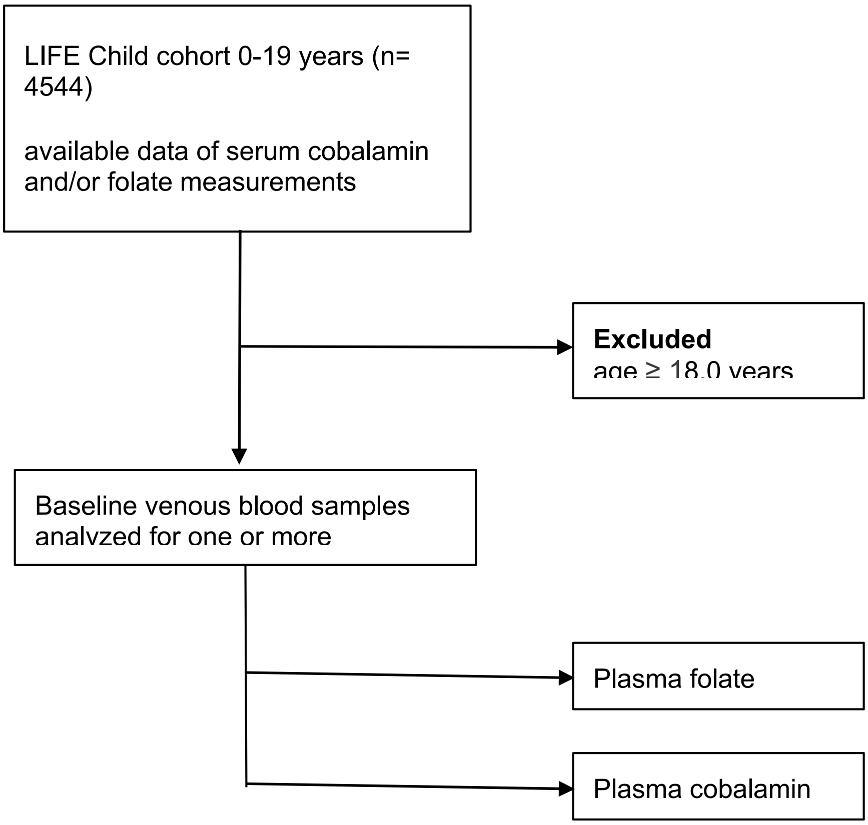

The flowchart indicates the inclusion and exclusion criteria for the study cohort. n = number of cases.

Table S2. Percentile distribution of plasma folate (nmol/l) as a function of age (0-18 years) derived from censored data based on the LIFE Child cohort of generally healthy children and adolescents (n=4171)

Sex	Age	P 2.5	P 10	P 50	P90	P 97.5	Mu	Sigma	Nu
Male	0.5	21.64	27.09	40.48	59.87	73.91	40.48	0.30	0.06
	1.0	20.12	25.21	37.70	55.82	68.95	37.70	0.31	0.06
	1.5	18.76	23.52	35.22	52.20	64.51	35.22	0.31	0.06
	2.0	17.60	22.07	33.09	49.09	60.70	33.09	0.31	0.06
	2.5	16.63	20.88	31.32	46.52	57.55	31.32	0.31	0.06
	3.0	15.86	19.92	29.92	44.48	55.06	29.92	0.31	0.06
	3.5	15.26	19.18	28.84	42.92	53.15	28.84	0.31	0.06
	4.0	14.80	18.61	28.02	41.73	51.72	28.02	0.31	0.06
	4.5	14.44	18.18	27.39	40.85	50.65	27.39	0.31	0.06
	5.0	14.17	17.84	26.91	40.17	49.84	26.91	0.31	0.06

	5.5	13.94	17.56	26.53	39.64	49.20	26.53	0.31	0.06
	6.0	13.74	17.33	26.20	39.19	48.68	26.20	0.31	0.06
	7.0	13.39	16.91	25.62	38.40	47.75	25.62	0.32	0.06
	8.0	13.02	16.46	25.00	37.56	46.75	25.00	0.32	0.06
	9.0	12.61	15.96	24.29	36.56	45.56	24.29	0.32	0.06
	10.0	12.16	15.40	23.50	35.45	44.23	23.50	0.32	0.06
	11.0	11.65	14.78	22.60	34.16	42.67	22.60	0.32	0.06
	12.0	11.03	14.02	21.49	32.55	40.70	21.49	0.32	0.06
	13.0	10.33	13.14	20.19	30.65	38.37	20.19	0.33	0.06
	14.0	9.62	12.26	18.87	28.71	35.99	18.87	0.33	0.06
	15.0	8.99	11.47	17.70	26.99	33.86	17.70	0.33	0.06
	16.0	8.45	10.79	16.70	25.52	32.05	16.70	0.33	0.06
	17.0	7.97	10.19	15.80	24.21	30.44	15.80	0.33	0.06
	18.0	7.54	9.66	15.01	23.04	29.01	15.01	0.33	0.06
Female	0.5	24.07	29.27	41.62	59.20	71.98	41.62	0.27	0.00
	1.0	22.43	27.31	38.91	55.44	67.49	38.91	0.27	0.00
	1.5	20.95	25.53	36.45	52.04	63.42	36.45	0.27	0.00
	2.0	19.66	23.98	34.30	49.07	59.86	34.30	0.27	0.00
	2.5	18.56	22.66	32.48	46.55	56.85	32.48	0.28	0.00
	3.0	17.65	21.58	30.99	44.51	54.41	30.99	0.28	0.00
	3.5	16.93	20.72	29.81	42.89	52.50	29.81	0.28	0.00
	4.0	16.36	20.05	28.90	41.67	51.05	28.90	0.28	0.00
	4.5	15.92	19.53	28.21	40.75	49.98	28.21	0.28	0.00
	5.0	15.57	19.12	27.68	40.06	49.19	27.68	0.28	0.00
	5.5	15.28	18.79	27.25	39.52	48.59	27.25	0.28	0.00
	6.0	15.03	18.50	26.89	39.07	48.09	26.89	0.29	0.00
	7.0	14.56	17.96	26.21	38.25	47.19	26.21	0.29	0.00
	8.0	14.07	17.40	25.50	37.36	46.19	25.50	0.29	0.00
	9.0	13.52	16.76	24.66	36.28	44.96	24.66	0.30	0.00
	10.0	12.90	16.03	23.69	35.01	43.48	23.69	0.30	0.00
	11.0	12.24	15.24	22.62	33.57	41.80	22.62	0.30	0.00
	12.0	11.58	14.45	21.54	32.10	40.06	21.54	0.31	0.00

13.0	10.95	13.70	20.52	30.71	38.42	20.52	0.31	0.00
14.0	10.40	13.04	19.61	29.49	36.98	19.61	0.31	0.00
15.0	9.91	12.46	18.82	28.42	35.74	18.82	0.32	0.00
16.0	9.46	11.92	18.09	27.44	34.59	18.09	0.32	0.00
17.0	9.02	11.40	17.37	26.47	33.45	17.37	0.32	0.00
18.0	8.61	10.91	16.70	25.57	32.38	16.70	0.33	0.00

n = number of measurements; P = percentile; Mu = location parameter; Sigma = spread parameter; Nu = skewness parameter

Table S3. Percentile distribution of plasma cobalamin (pmol/l) as a function of age (0-18 years) based on the LIFE Child cohort of generally healthy children and adolescents (n=4229)

Sex	Age	P 2.5	P 10	P 50	P90	P 97.5	Mu	Sigma	Nu
Male	0.5	132.98	174.47	297.50	522.44	713.15	297.50	0.43	-0.10
	1	147.53	192.52	322.95	552.76	741.09	322.95	0.41	-0.07
	1.5	163.02	211.59	349.47	584.39	771.22	349.47	0.40	-0.05
	2	178.89	230.99	376.11	616.16	802.19	376.11	0.38	-0.03
	2.5	194.43	249.88	401.74	646.59	832.20	401.74	0.37	0.00
	3	208.97	267.43	425.20	674.03	859.17	425.20	0.36	0.01
	3.5	221.85	282.85	445.37	696.94	881.21	445.37	0.35	0.03
	4	232.50	295.41	461.26	714.01	896.79	461.26	0.34	0.04
	4.5	240.43	304.55	472.14	724.48	905.16	472.14	0.34	0.05
	5	245.32	309.91	477.67	728.09	906.18	477.67	0.33	0.06
	5.5	247.31	311.67	478.14	725.33	900.43	478.14	0.33	0.06
	6	246.90	310.43	474.41	717.38	889.29	474.41	0.33	0.06
	7	242.40	303.27	460.48	694.27	860.43	460.48	0.32	0.04
	8	236.39	294.20	444.02	668.71	829.87	444.02	0.32	0.01
	9	229.71	284.39	426.42	640.87	795.88	426.42	0.32	-0.01
	10	221.09	272.58	406.36	608.91	755.88	406.36	0.31	-0.03
	11	208.38	256.91	383.17	574.76	714.05	383.17	0.31	-0.04
	12	191.93	237.73	357.20	538.84	670.94	357.20	0.32	-0.02
	13	175.56	218.88	332.06	503.82	628.27	332.06	0.33	0.00
	14	163.64	205.01	312.74	475.00	591.60	312.74	0.33	0.02
	15	157.15	197.07	300.22	453.52	562.34	300.22	0.33	0.05
	16	154.78	193.75	293.43	439.32	541.55	293.43	0.32	0.07
	17	155.17	193.61	290.84	430.94	527.89	290.84	0.31	0.09

	18	156.76	194.78	289.84	424.66	516.77	289.84	0.30	0.10
Female	0.5	129.08	174.53	310.81	558.85	765.44	310.81	0.45	-0.03
	1	146.71	194.85	334.90	579.86	777.80	334.90	0.43	-0.02
	1.5	165.53	216.26	359.81	601.94	792.22	359.81	0.40	-0.02
	2	184.97	238.10	384.74	624.09	807.52	384.74	0.38	-0.02
	2.5	204.27	259.51	408.64	645.09	822.29	408.64	0.36	-0.01
	3	222.46	279.43	430.32	663.55	834.97	430.32	0.34	-0.01
	3.5	238.60	296.88	448.71	678.38	844.39	448.71	0.32	0.00
	4	251.90	311.03	463.03	688.88	849.89	463.03	0.31	0.00
	4.5	261.84	321.41	472.91	694.87	851.39	472.91	0.30	0.01
	5	268.22	327.86	478.34	696.52	849.16	478.34	0.29	0.01
	5.5	271.21	330.56	479.57	694.16	843.51	479.57	0.29	0.02
	6	271.20	329.97	477.12	688.34	834.97	477.12	0.29	0.02
	7	264.67	321.65	464.45	669.87	812.81	464.45	0.29	0.01
	8	252.37	307.02	445.05	645.97	787.21	445.05	0.29	-0.01
	9	237.32	289.46	422.74	620.32	761.43	422.74	0.30	-0.03
	10	222.85	272.88	402.53	598.73	741.36	402.53	0.31	-0.05
	11	209.83	258.41	386.05	583.10	728.74	386.05	0.32	-0.07
	12	197.94	245.63	372.69	572.62	722.64	372.69	0.33	-0.07
	13	186.10	232.84	359.01	561.01	714.63	359.01	0.34	-0.07
	14	173.57	218.45	340.83	539.46	692.11	340.83	0.35	-0.07
	15	161.40	203.50	319.10	508.78	655.99	319.10	0.36	-0.08
	16	151.24	190.38	298.56	478.32	619.64	298.56	0.36	-0.10
	17	143.39	179.97	281.94	454.51	592.82	281.94	0.36	-0.13
	18	136.87	171.27	268.23	436.10	573.97	268.23	0.36	-0.17

n = number of measurements; P = percentile; Mu = location parameter; Sigma = spread parameter; Nu = skewness parameter

Figure S2. (a) Cobalamin, (b) folate according to BMI

(a)

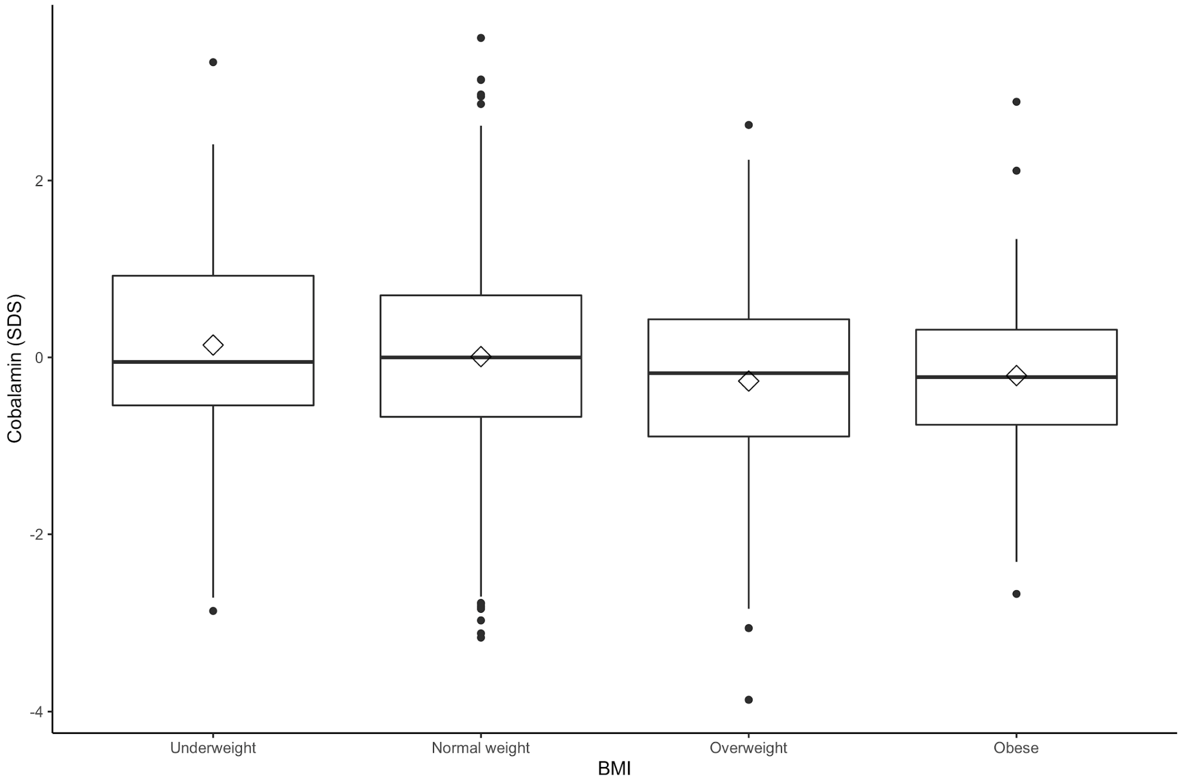

(b)

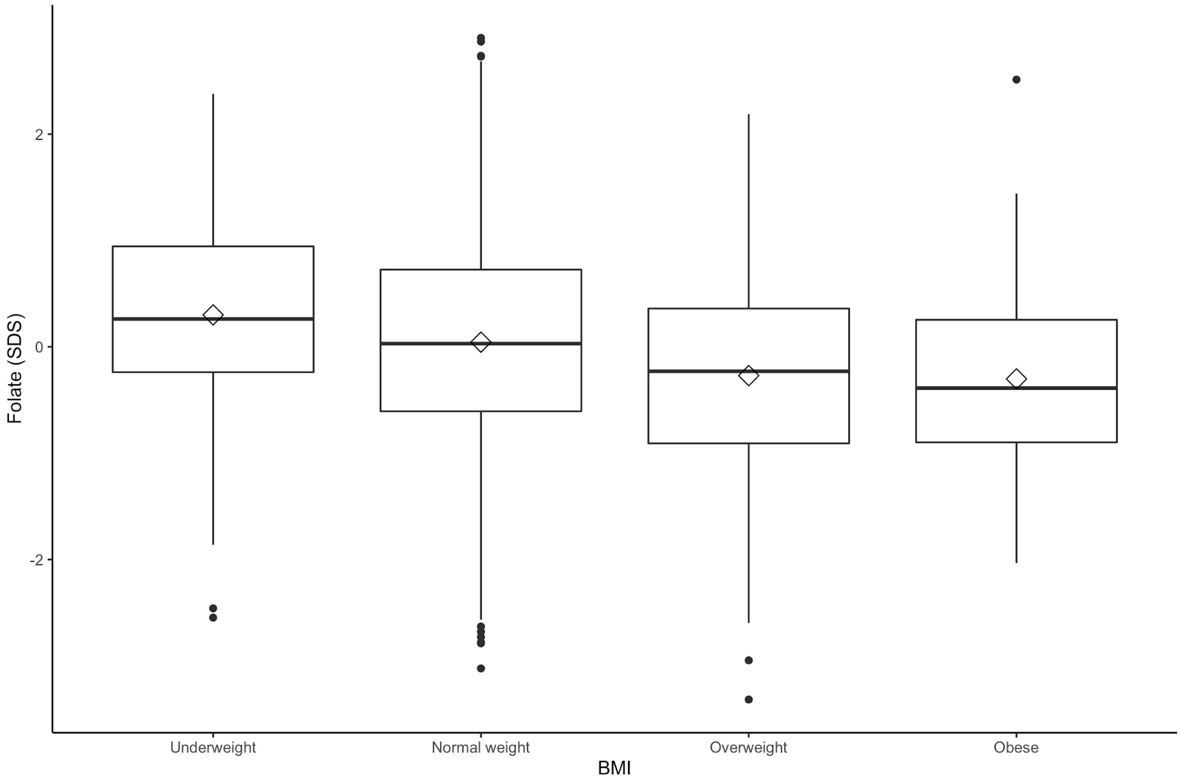

Higher BMI was significantly associated with lower serum vitamin concentrations ($p < 0.001$). Mean (square shape); 25th, 50th, 75th percentile are depicted. BMI-SDS groups: underweight: BMI-SDS < -1.28; normal weight: BMI-SDS -1.28 to < 1.28; overweight: BMI-SDS >1.28 to 1.88; obese: BMI-SDS >1.88. SDS = standard deviation score; BMI = body mass index.

Figure S3. Folate according to SES

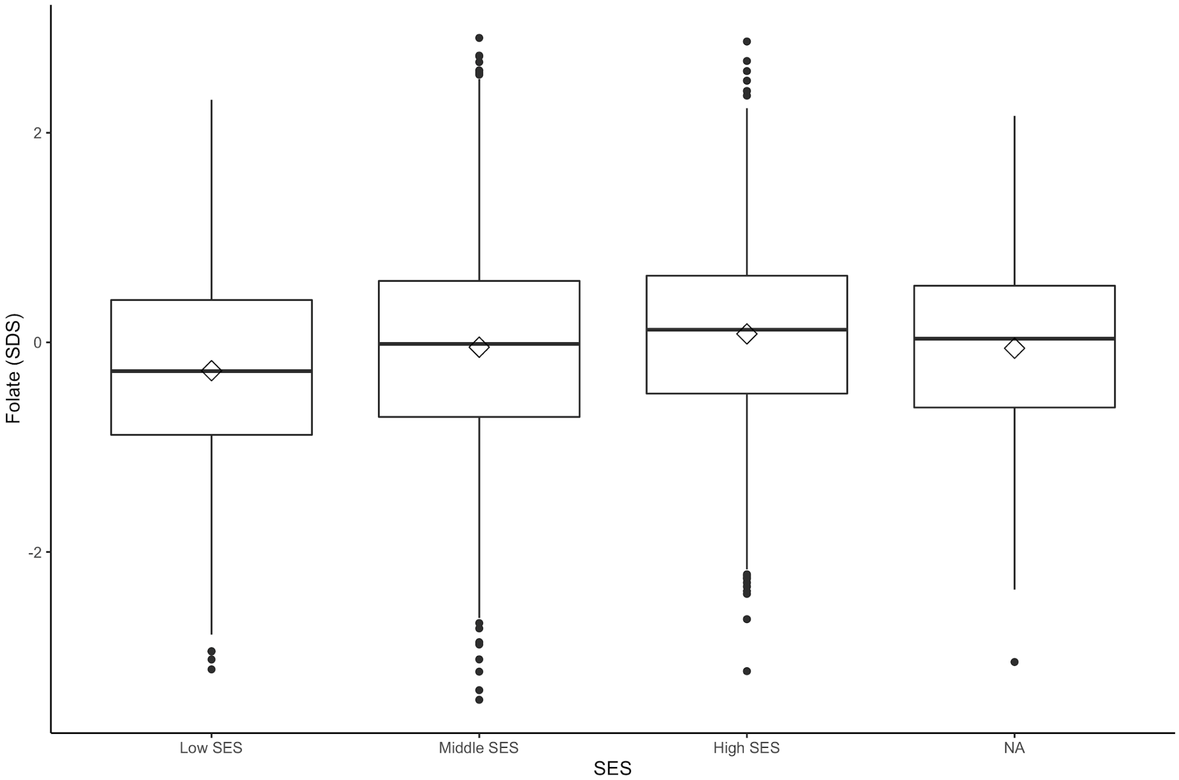

Higher SES was significantly associated with higher serum folate concentrations. SES groups: low: WI 3-8.4; middle: WI 8.5-15; high: 15.5-21. SES = socioeconomic status; WI = Winkler Index; SDS = standard deviation score.
